# From circuits to behavior: Amygdala dysfunction in fragile X syndrome

**DOI:** 10.3389/fnint.2023.1128529

**Published:** 2023-03-09

**Authors:** Matthew N. Svalina, Regina Sullivan, Diego Restrepo, Molly M. Huntsman

**Affiliations:** ^1^Medical Scientist Training Program, University of Colorado Anschutz Medical Campus, Aurora, CO, United States; ^2^Neuroscience Graduate Program, University of Colorado Anschutz Medical Campus, Aurora, CO, United States; ^3^Department of Pharmaceutical Sciences, University of Colorado Anschutz Medical Campus, Aurora, CO, United States; ^4^Department of Cell and Developmental Biology, University of Colorado Anschutz Medical Campus, Aurora, CO, United States; ^5^Brain Institute, Nathan Kline Institute, Orangeburg, NY, United States; ^6^Child and Adolescent Psychiatry, Child Study Center, New York University School of Medicine, New York, NY, United States; ^7^Department of Pediatrics, University of Colorado Anschutz Medical Campus, Aurora, CO, United States

**Keywords:** critical period, synaptic plasticity, basolateral amygdala, fragile X syndrome, development, E/I balance

## Abstract

Fragile X syndrome (FXS) is a neurodevelopmental disorder caused by a repeat expansion mutation in the promotor region of the *FMR1* gene resulting in transcriptional silencing and loss of function of fragile X messenger ribonucleoprotein 1 protein (FMRP). FMRP has a well-defined role in the early development of the brain. Thus, loss of the FMRP has well-known consequences for normal cellular and synaptic development leading to a variety of neuropsychiatric disorders including an increased prevalence of amygdala-based disorders. Despite our detailed understanding of the pathophysiology of FXS, the precise cellular and circuit-level underpinnings of amygdala-based disorders is incompletely understood. In this review, we discuss the development of the amygdala, the role of neuromodulation in the critical period plasticity, and recent advances in our understanding of how synaptic and circuit-level changes in the basolateral amygdala contribute to the behavioral manifestations seen in FXS.

## Introduction

Fragile X syndrome (FXS) is a rare X-linked recessive genetic disorder affecting approximately 1:5,000 males and 1:4,000–8,000 females (Hagerman et al., 2017). The physical phenotype of individuals with FXS is characterized by large ears, long narrow faces, hyperextensible joints, and macroorchidism (Kidd et al., 2014). The clinical features of FXS include a variety of neurologic and psychiatric disorders including intellectual disability, attention deficit hyperactivity disorder (ADHD), anxiety, social avoidance, increased incidence of seizures and epilepsy, autism spectrum disorders (ASDs), and hypersensitivity to sensory stimuli (Hagerman et al., 2009).

Fragile X syndrome is caused by a CGG repeat expansion in the 5′ untranslated region (UTR) of the fragile X messenger ribonucleoprotein 1 (*FMR1*) gene located at Xq27.3. Individuals with trinucleotide repeats numbering < 50 are phenotypically normal and without clinical symptomatology. Carriers of the premutation repeat expansion (numbering between 55 and 200) exhibit the distinct fragile X associated-tremor ataxia syndrome (FXTAS). FXTAS is an adult-onset neurodegenerative disorder characterized by intention tremor, parkinsonism, and generalized brain atrophy (Bale et al., 2010). The pathophysiology of FXTAS has been shown to result in part from excessive production of CGG repeat expanded *FMR1* messenger RNA (mRNA) (Bale et al., 2010). This excessive mRNA production has been shown to be neurotoxic *in vitro* and *in vivo* (Jin et al., 2003; Hukema et al., 2014; Swinnen et al., 2020) resulting in ubiquitin and FMRpolyG-positive intranuclear inclusions and subsequent cerebellar Purkinje cell loss (Buijsen et al., 2014). Trinucleotide repeat expansions (numbering > 200) result in hypermethylation of the promoter region of the *FMR1* locus and subsequent transcriptional silencing of the *FMR1* gene (Fu et al., 1991). Functionally, and in contrast to FXTAS, this results in the absence of the fragile X messenger ribonucleoprotein 1 protein (FMRP) (Verkerk et al., 1991). The clinical severity of FXS depends in large part on the number of trinucleotide repeat expansions in the 5′ UTR of the *FMR1* gene and on cell-autonomous FMRP production (Hagerman et al., 2017).

Since the identification of the molecular pathophysiology underpinning FXS (Fu et al., 1991; Verkerk et al., 1991), subsequent investigations have elucidated the functional role of the FMRP. The FMRP is an RNA binding protein with a known role in regulating mRNA translation (Chen et al., 2003; Darnell et al., 2011) and is ubiquitously expressed throughout the brain and present in all neuronal cell compartments (Christie et al., 2009). FMRP binds directly to translational machinery and induces ribosomal stalling with suppression of protein synthesis (Darnell et al., 2011). However, the regulatory role of FMRP in protein synthesis is complex and varied. FMRP regulates many target genes involved in neuronal synapse formation and maintenance and plasticity responses (Casingal et al., 2020). Loss of FMRP results in the dysregulated synthesis of proteins essential for normal corticogenesis, cellular and synaptic function, and synaptic plasticity (Sidorov et al., 2013; Sears and Broadie, 2017). In addition to its role in translation suppression, new evidence is emerging that FMRP may enhance, rather than repress, protein translation and that loss of FMRP induces the down-regulation of target genes implicated in ASDs and neurodevelopmental disorders (NDDs) (Greenblatt and Spradling, 2018). Thus, FXS may share pathology common to many different forms of ASDs. Indeed, the *Fmr1*KO mouse model of FXS not only recapitulates many aspects of the human condition but has been a key tool for understanding ASDs more broadly.

### FMRP-associated cellular, synaptic, and circuit dysfunction in FXS

At the cellular level, loss of FMRP affects ion channel function with profound effects on membrane properties and neuronal intrinsic excitability beyond mRNA translational modulation (Contractor et al., 2015). Importantly, this non-canonical function of FMRP appears to involve modulation of ion channels via direct FMRP binding and modulation of channel kinetics (Kshatri et al., 2020). For example, FMRP has been shown to regulate the function of numerous potassium channels [including K_v_3.1 (Darnell et al., 2011), K_v_4.2 (Gross et al., 2011), HCN (Brager et al., 2012; Zhang et al., 2014; Brandalise et al., 2020), and BK (Torres et al., 2007; Deng et al., 2013), Slack (Brown et al., 2010)] and calcium channels [Ca_v_2.2 (Ferron et al., 2014)] (Ferron, 2016). FMRP-mediated potassium channel dysfunction has been shown to underpin membrane hyperexcitability and increased UP states in layer 4 somatosensory barrel cortex principal neurons (PNs) (Gibson et al., 2008). Further, alterations in BK and h- channel function has been shown to contribute to sensory hypersensitivity by altering a number of membrane properties including resting membrane potential and dendritic excitability in S1 excitatory PNs in the period immediately following the somatosensory critical period (CP). Broadly this results in increased action potential (AP) output and integration of synaptic input (Zhang et al., 2014). Loss of FMRP also results in abnormal Slack function and decreases in late after-hyperpolarization which enables repetitive firing in PNs in *Fmr1*KO mice.

At the level of the synapse, FMRP-driven dysfunction in Ca_v_2.2 leads to alterations in presynaptic neurotransmitter release in dorsal root ganglion (DRG) neurons (Ferron et al., 2014). FMRP is upregulated by group 1 (Gp1) metabotropic glutamate receptors (mGluRs) following post-synaptic activation (Weiler et al., 1997). In particular, loss of FMRP leads to increased expression of mGluRs as well as increases in α-amino-3-hydroxy-5-methyl-4-isoxazolepropionic acid (AMPA) receptor turnover (Bear et al., 2004). This AMPA receptor turnover has been shown to mediate mGluR-mediated long-term depression (LTD) in hippocampal neurons following post-synaptic stimulation of excitatory principal neurons (PNs) in older animals (Weiler and Greenough, 1993; Weiler et al., 1997). Altered long-term potentiation (LTP) and LTD have also been observed in the cingulate cortex, visual cortex, and the amygdala (Bhakar et al., 2012). As with ion channel dysfunction, plasticity alterations seen in FXS may be region and developmental stage-specific. For example, in contrast to later life mGluR-LTD in the hippocampus, early life enhancements in synaptic plasticity have been observed in hippocampal neurons of *Fmr1*KO mice (Gray et al., 2019). This effect may be in part mediated by increased mGluR-mediated expression of R-voltage dependent calcium channel (R-VDCC)-mediated Ca2+ spikes (Gray et al., 2019). Thus, numerous FMRP-mediated channelopathies contribute not only to altered intrinsic membrane properties and hyperexcitability but synaptic and circuit-level dysfunction as well.

In addition to cellular, circuit, and plasticity alterations in excitatory PNs in FXS, loss of FMRP also has a known role in interneuron (IN) dysfunction as well (Paluszkiewicz et al., 2011b; Cea-Del Rio and Huntsman, 2014; Martin et al., 2014). FMRP is widely expressed in inhibitory INs (Olmos-Serrano et al., 2010) and many functional elements of γ-aminobutyric acid (GABA) neurotransmission are dysregulated in the context of loss of FMRP (Paluszkiewicz et al., 2011a). As early as P5, GABA concentrations are altered in the frontal cortex and thalamus in *Fmr1*KO mice (Reyes et al., 2020). Functional GABA_A_ receptors are heteropentamers whose unique receptor subunit composition determines their pharmacologic and physiologic properties (Hevers and Luddens, 1998; Rudolph and Mohler, 2006). Loss of FMRP leads to reductions in synaptic and extrasynaptic GABA_A_ receptors with concomitant reductions in GABA_A_ α, β, γ receptor subunit expression (D’Hulst et al., 2006; Gantois et al., 2006; Huang and Richter, 2007). Significant alterations have also been found in the biochemical pathways necessary for the synthesis of GABA including alterations in the GABA synthesizing enzyme GAD65 (D’Hulst et al., 2009; Adusei et al., 2010; Olmos-Serrano et al., 2010). Further, dysfunction in the systems necessary for GABA catabolism, reuptake mechanisms, and subcellular localization of GABA_A_ receptors have also been observed (D’Hulst et al., 2009; Adusei et al., 2010). Thus, marked IN dysfunction is a contributing factor to multiple hyperexcitable phenotypes observed in FXS (Contractor et al., 2015). Importantly, these alterations are highly region- and developmental timepoint specific (Paluszkiewicz et al., 2011a). Taken together, these data demonstrate that the loss of function of FMRP has wide-ranging effects throughout the brain at all developmental ages but these effects may be most pronounced during early development.

### Critical period dysfunction in FXS

Fragile X syndrome is a NDD in which the dysregulation of protein synthesis results in significant deficits in neuronal, synaptic, and circuit function during critical periods of development (Contractor et al., 2015). Critical periods are stages in early brain development in which precise cellular, synaptic, and experience-dependent input is necessary for the proper development of neural circuits and systems (Hensch, 2005). In turn, CP plasticity is critically dependent on the precise balance of excitation and inhibition (E/I) during normal synaptogenesis. Interestingly, CP plasticity has been shown to be dependent on the maturation of GABAergic neurotransmission (Fagiolini and Hensch, 2000; Iwai et al., 2003; Fagiolini et al., 2004).

For example, the onset of the visual cortex CP has been shown to correspond with the developmental emergence of parvalbumin-expressing (PV+) inhibitory INs (Del Rio et al., 1994) around post-natal (PN) day 14. Indeed, precocious emergence of CP opening is mediated by brain-derived neurotrophic factor (BDNF)-driven enhancements of PV+ GABAergic neurotransmission (Huang et al., 1999). An accelerated post-natal rise of BDNF in the visual cortex of transgenic mice has been shown to mediate a precocious opening of CP plasticity such that increased ocular dominance plasticity (ODP) leads to an early-life increase in visual acuity. Importantly, this was driven by robust maturation of GABAergic circuitry (Huang et al., 1999). Within the CP for the visual system, ODP following lid suture requires homeostatic scaling of inhibition mediated by PV+-fast spiking cells. In particular, reductions in glutamatergic neurotransmission in the visual cortex following lid suture are accompanied by reductions in feed-forward inhibitory input onto excitatory PNs. This process is mediated by reduced excitatory input onto PV+ cells. Further, pharmacologically enhancing inhibition during lid suture reduces ODP, and reduction of PV+-firing via pharmacogenetic approaches extends ODP (Kuhlman et al., 2013). Additionally, somatostatin-expressing (SOM+) INs also exhibit enhanced excitability during the CP implicating dendritic-targeting inhibition from SOM+ as an important factor in ODP (Lazarus and Huang, 2011). This precise synaptic scaling and homeostatic plasticity has been shown to be impaired in FXS (Zhang et al., 2018).

In FXS, E/I imbalance results in aberrant CP plasticity across diverse brain regions including the somatosensory cortex and the basolateral amygdala (BLA) (Rubenstein and Merzenich, 2003; Nelson and Valakh, 2015). Indeed, alterations in CP plasticity are thought to underpin the temporal manifestation of neurologic and psychiatric deficits observed during early childhood development—known as sensitive time windows (Meredith et al., 2012; Meredith, 2015). In the somatosensory cortex, the period of programed synaptic and cellular changes required for normal circuit development and refinement is shorter in duration and occurs later in development (Harlow et al., 2010). Anatomically, *Fmr1*KO mice exhibit altered dendritic spine morphology and spine turnover in the barrel cortex at P14 (Cruz-Martin et al., 2010). At the synaptic level, glutamatergic projections in the barrel cortex of *Fmr1*KO mice have been found to exhibit lower connection probabilities and diffuse axonal arbors accompanying decreases in experience-dependent plasticity at P14 (Bureau et al., 2008). Further, *Fmr1*KO animals show an early life increase in the N-methyl-d-aspartate (NMDA)/AMPA ratio and an increase in NMDA-silent synapses during the barrel cortex CP (Harlow et al., 2010). Experience-dependent plasticity during critical periods involves the conversion of previously formed NMDA-silent synapses via up the upregulation of AMPARs. This upregulation of AMPARs is thought to underlie the unique changes seen in neonatal rodent olfactory learning and odor preference (Franks and Isaacson, 2005; Opendak et al., 2018). NMDA receptor activation and spike-timing dependent plasticity (STDP) are also altered in *Fmr1*KO mice where LTD at cortical synapses is robust, whereas LTP diminished (Desai et al., 2006). However, excitatory projections resemble that of the wildtype animal by the third week of life suggesting that homeostatic mechanisms may shift developmental trajectories in the *Fmr1*KO barrel cortex (Bureau et al., 2008).

Additionally, differences in BDNF signaling have been observed in the barrel cortex and in the hippocampus of *Fmr1*KO mice at P14 (Louhivuori et al., 2011). Increased BDNF signaling has been observed in the hippocampus whereas decreased BDNF signaling was observed in the somatosensory cortex (Louhivuori et al., 2011). Thus, regional differences in BDNF signaling may underpin early life plasticity alterations with distinctly different effects in the hippocampus and somatosensory cortex (Louhivuori et al., 2011; Gray et al., 2019).

Depolarizing GABA has been shown to play a pivotal role in cell migration, synapse formation, and integration in early development (Ge et al., 2006). Interestingly, GABA has been shown to be depolarizing as late as P13 in the somatosensory cortex (He et al., 2019). This delay in the GABA polarity switch is driven by developmental alterations in the regulation of the chloride transporters NKCC and KCC2 (He et al., 2014). Recent work examining the GABA polarity switch in a schizophrenia-risk gene model demonstrated that excessive PV+ inhibitory tone after the polarity switch drives excessive glutamatergic synapse formation further exacerbating E/I imbalance (Kang et al., 2019). Interestingly, in conditional deletion models of ASDs, peripheral sensory dysfunction arose from PV+ interneuron dysfunction in both the BLA and the barrel cortex as early as P5. Thus, distinct behavioral deficits, including increased tactile sensitivity and anxiety, emerged in an early post-natal time deletion-specific manner. Further, treatment in early life with GABA_A_ receptor agonists improved somatosensory hyperreactivity and anxiety in these animals (Orefice et al., 2019). Thus, prolongation of the chloride reversal potential may enhance excitatory synapse function in *Fmr1*KO mice and this effect may be mediated by PV+ INs.

### Development of the basolateral nuclei of the amygdaloid complex

The amygdaloid complex is a medial temporal lobe structure generally considered to have two major divisions, corticomedial and basolateral (Hopkins, 1975). The basolateral division is composed of the lateral (LA) and basal (BA) nuclei whose glutamatergic progenitors have their embryologic origin in the ventral pallium. GABAergic progenitors have their origin in the subpallium (medium ganglionic eminence) (Waclaw et al., 2010). Together, these nuclei form the BLA and have an essential function in the processing of emotional information and assigning valance to emotional stimuli (Benarroch, 2015). Further, the BLA is the nuclei responsible for conditioned learning of aversive stimuli (Blair et al., 2005). Anatomical studies in rabbits have demonstrated that during the first three post-natal weeks of development cerebral volume and neuronal density increases (reaching mature levels by P60 and P30, respectively). Further, acetylcholinesterase activity increases over this time indicating that the neuromodulatory actions of acetylcholine may play an important role in CP plasticity. Importantly, these morphological changes are similar in the rodent BLA (Jagalska-Majewska et al., 2003; Ryan et al., 2016).

From birth to adulthood, PNs in the rodent BLA during the first three post-natal weeks undergo a number of morphological changes including an increase in the size of the soma, maturation of dendritic arbors around peri-weaning age, and an increase in the dendritic spine density until age P60 (Ryan et al., 2016). The neuronal density in the BLA is completely established by P7 (Berdel et al., 1997). In turn, a number of intrinsic electrophysiological parameters mature in PNs in the BLA. Specifically, AP frequencies increase, input resistances decrease, and spike amplitudes increase with age from P7 to P60 (Ryan et al., 2016). Maturational and physiologic changes in PNs in the BLA during the first two post-natal weeks accompany synaptic and microcircuit development. By the second post-natal week, the thalamic afferents are completely established by P9, followed by establishment of afferents from the pre-frontal cortex by P14 (Bouwmeester et al., 2002). Functional resting state MRI has revealed that the neonatal brain exhibits undifferentiated connections to both primary sensory and cortical areas compared to the adult brain. Thus, experience-dependent plasticity likely influences cortical connectivity (Hansen et al., 2020). To that end, the emergence of cue fear-learning correlates with the developmental emergence of LTP at cortical and thalamic afferents occurring at approximately P10 (Berdel et al., 1997), with the complexity of learning features increasing with the maturation of connectivity between the BLA and other brain areas (Hunt et al., 2007; Li et al., 2012).

### Neuromodulatory input to the basolateral amygdala

A number of neuromodulators have important roles in the modulation of fear learning. Neuromodulators are important regulators of circuit output playing critical roles in regulating neuronal excitability, pre-synaptic release of neurotransmitters, LTD and potentiation, and mediation of brain state changes that underlie behavior (Nadim and Bucher, 2014). Neuromodulatory systems also have well-known roles in CP induction and cortical plasticity (Herlenius and Lagercrantz, 2004; Hensch, 2005; Larsen and Luna, 2018). Indeed, neuromodulators are employed throughout the forebrain with distinct effects on synaptic transmission and behavior.

#### Cholinergic input

The BLA receives dense cholinergic input from a number of basal forebrain structures including the nucleus basalis and the horizontal limb of the diagonal band of Broca and the magnocellular preoptic area. In the rodent, cholinergic afferents in the BLA mirror those found in the human including the horizontal diagonal band, magnocellular preoptic nucleus, and ventral pallidum (Woolf, 1991). In the rodent, cholinergic input to the BLA is also contributed by the lateral preoptic area, medial septal nucleus, bed nucleus of the stria terminalis, and substantia innominata (Woolf and Butcher, 1982). However, the majority of cholinergic input originates from the magnocellular neurons in the nucleus basalis of Meynert (NBM) (Kitt et al., 1994). Evidence for the effects of cholinergic neuromodulation on plasticity and associative behaviors is extensive. Cholinergic actions on forebrain targets have been implicated in arousal (Jones, 2008), memory (Croxson et al., 2011), attention, and learning (Ljubojevic et al., 2018). Within the BLA, cholinergic signaling modulates fear-learning via increases in PN excitability and the enhancement of LTP forming durable fear memories resistant to extinction (Jiang et al., 2016).

#### Noradrenergic input

The main source of noradrenergic innervation in the BLA is the locus coeruleus (LC). The LC is located within the pons of the brainstem and is comprised of three fiber projections the ascending pathway, the cerebellar pathway, and the descending pathway. The ascending pathway innervates structures within the midbrain including the limbic system (Szabadi, 2013). Within the amygdaloid complex dense innervation has been noted within the basal and central nuclei (Jones and Moore, 1977; Fallon et al., 1978). Indeed, there exists a specific population of neurons within the LC that project (LA-projecting) to the LA whose activation is necessary for fear-learning (Uematsu et al., 2017). The principal neurotransmitter, norepinephrine, mediates its effects in the BLA via functionally-distinct adrenergic receptors: α1, β1, and β2-receptors mediate excitation of PNs within the BLA and α2 receptors mediate inhibition. Within the BLA, the α1 receptor is highly expressed within the LA, with modest expression noted in the central amygdala (CeA) (Jones and Moore, 1977; Pieribone et al., 1994). Interestingly, a recent study noted high co-expression of α1 with glutamic acid decarboxylase (GAD), GABA, and NMDA receptors (Papay et al., 2006). This suggests that α1-receptors not only mediate an excitatory effect on PNs but may also enhance GABAergic signaling via a direct effect on inhibitory interneurons. Administration of an α1-adrenergic antagonist to mice during fear-conditioning showed similar levels of freezing to control animals. However, α1-adrenergic antagonist-treated mice exhibited higher levels of post-training extinction (Favero et al., 2018).

Expression of α2-receptors is confined mainly to the CeA with only low-level expression noted in the lateral and basal nuclei (Pascual et al., 1992; Glass et al., 2002). This suggests that the predominate effects of α2-adrenergic activity in the central output nucleus of the amygdaloid complex may be to mediate the autonomic and motor responses to fear and anxiety. Within the amygdaloid complex, moderate β-receptor expression is highest in the cortical nuclei with moderate expression noted within the CeA (Wanaka et al., 1989). Higher levels of expression of β1 and β2 receptors have been observed in the BLA (Rainbow et al., 1984). High densities of β-receptors have also been observed in the human amygdala post-mortem (Pazos et al., 1985). The role of β-receptors in the pathology of fear and anxiety is well-described (AlOkda et al., 2019). β-receptors have been found to enhance fear potentiation and β-receptor antagonists are capable of enhancing fear extinction (Debiec et al., 2011). Furthermore, administration of the β-adrenergic antagonist propranolol abolishes amygdala-associated memory enhancement (Strange and Dolan, 2004).

The developmental effects of noradrenergic neuromodulation in the BLA are just beginning to be studied with a recent study identifying an age-dependent effect of adrenergic agonism on increased PN spike frequency in juvenile mice but not in adult mice. This effect was mediated via the downregulation of tonic inhibition in juvenile animals (Fink and LeDoux, 2018). Norepinephrine (NE) is also known to potentiate associative learning at thalamo-amygdala synapses (Tully et al., 2007) and is capable of suppressing feed-forward inhibition (FFI) (Bergles et al., 1996; Ehrlich et al., 2009). Similarly, NE has also been demonstrated to play a distinct role in the conversion of eligibility traces for LTP (Elwood and Keeling, 1982). Thus, noradrenergic neuromodulation is necessary for state-dependent neural coding and threat learning.

#### Serotonergic input

The BLA receives serotonergic innervation mainly from the nucleus raphe dorsalis found in the brainstem. A total of 10% of serotonergic efferent projections throughout the forebrain are known to innervate the BLA (Bobillier et al., 1976; Ma et al., 1991; Bonn et al., 2013). The actions of serotonin in the BLA are considerably more complex given the diversity of serotonergic receptors and their myriad actions. During Pavlovian fear-conditioning, serotonin is known to increase in concentration in the BLA (Zanoveli et al., 2009). Serotonin primarily affects GABAergic INs via the 5-HT2A receptor enhancing inhibitory neurotransmission and FFI (Rainnie, 1999; Jiang et al., 2009). However, following acute stress, downregulation of the 5-HT2A receptor on INs decreases FFI (Jiang et al., 2009). Indeed, reductions in serotonergic tone have been shown to decrease GABAergic FFI in the BLA and have been postulated to underpin anxiety and fear disorders (Wang et al., 2019). However, studies evaluating the effects of serotonin on fear learning have produced conflicting results. Rodents treated with a selective serotonin reuptake inhibitor (SSRI) prior to fear-conditioning exhibited increased contextual and cued fear (Bosker et al., 2001). In contrast, under conditions of depleted serotonin, rodents exhibited decreased fear acquisition (Dobyns et al., 1984). Thus, the exact role that serotonin plays in fear acquisition remains unclear, but these studies suggest that it may be receptor subtype specific.

#### Dopaminergic input

The BLA receives dopaminergic input primarily from the ventral tegmental area (VTA) and substantia nigra (Asan, 1997). Dopaminergic projections innervate both PNs (Brinley-Reed and McDonald, 1999) and INs (Pinard et al., 2008) and have distinct effects based on cell-type specific receptor expression. Principal neurons in the BLA have been found to express D1 with dopamine-enhancing PN excitability (Kroner et al., 2005). The D2 receptor is found on PV+ INs and functions to suppress GABA release at the synapse (Bissiere et al., 2003; Chu et al., 2012). The role of dopaminergic neuromodulation in aversion learning is well-described. In rodents, fear-potentiated startle is enhanced upon electrical stimulation of the VTA (Borowski and Kokkinidis, 1996). Conversely, administration of D1-receptor antagonists decreases fear-potentiated startle and freezing behavior following Pavlovian conditioning (Nader and LeDoux, 1999; Greba and Kokkinidis, 2000). In odor-cued fear-conditioning paradigms, administration of haloperidol decreased PN excitability and excitatory synaptic strengthening following odor-shock conditioning (Rosenkranz and Grace, 2002b). Thus, the overall net effect of dopaminergic neuromodulation is to increase PN excitability and to suppress IN-mediated FFI in micro-circuits important for fear-learning (Rosenkranz and Grace, 1999, 2002a; Bissiere et al., 2003). Interestingly, dopamine suppression underpins the maternal attenuation of infant fear-learning during a sensitive period in the development of the BLA (Barr et al., 2009; Opendak et al., 2019).

### Amygdala dysfunction in FXS

The limbic system, and in particular the BLA, has been implicated in a variety of neuropsychiatric disorders including anxiety, panic disorder, post-traumatic stress disorder, and substance use disorder (Martin et al., 2010; Janak and Tye, 2015). Individuals with FXS and ASDs exhibit an increased prevalence of socioemotional problems including social anxiety, social withdrawal, hyperactivity, and gaze aversion (Bregman et al., 1988; Cohen et al., 1988; Hagerman et al., 1991; Cordeiro et al., 2011; Tonnsen et al., 2013). However, structural neuroimaging studies examining volume differences in individuals with FXS have been inconsistent. Small studies and case reports have documented neuroanatomical increases in amygdala volume in individuals with FXS (Reiss et al., 1995), whereas larger studies have found no difference or reduced amygdala volumes (Kates et al., 1997; Gothelf et al., 2008; Hazlett et al., 2009). One recent volumetric study identified persistent developmental enlargements of caudate volumes in children with FXS as early as 6 months of age but no differences in amygdala volume. In contrast, children with ASDs exhibited rapidly changing amygdala volumes over the first 2 years of life. Interestingly, these changes were observed prior to behavioral symptom onset and diagnosis demonstrating that developmental trajectories become altered very early in life (Shen et al., 2022). From a functional connectivity standpoint, individuals with FXS have been shown to exhibit aberrant white matter integrity and interconnectivity (Barnea-Goraly et al., 2003), with reduced integration of subcortical regions including the amygdala, anterior cingulate, and temporal pole (Bruno et al., 2017). Taken together, these data suggest that structural neuroanatomy may not fully account for the behavioral differences observed in FXS.

Functional imaging studies have identified a number of key differences in amygdala responses in FXS. Pronounced gaze aversion with abnormal gaze processing has been observed in children and young adults with FXS (Garrett et al., 2004). However, no differences in amygdala activation were observed between neurotypical and FXS patients during facial-emotion judgment or in the presentation of fearful stimuli (Hagan et al., 2008; Holsen et al., 2008). In contrast, children with FXS have been found to exhibit increased autonomic responses to fearful stimuli in an age-dependent manner (Tonnsen et al., 2013). Comparison of these studies is confounded by differences in experimental design, experimental group parameters, and the type of behavioral task employed. Thus, the precise neural correlates of amygdala dysfunction in FXS patients remain unclear.

At the cellular level, hyperexcitability within the BLA has been implicated in the pathophysiology of amygdala-based behaviors (Bulow et al., 2022). Our own work has identified distinct developmental alterations in the hyperexcitability of PNs in the BLA including profound changes in intrinsic firing rates and low threshold spiking activity (Svalina et al., 2021, 2022). At the circuit level, in adult mice, our group and others have identified profound alterations in GABAergic neurotransmission including deficits in phasic and tonic inhibition, GABA_A_ receptor composition, and GABA synthesis (Olmos-Serrano et al., 2010; Paluszkiewicz et al., 2011a; Cea-Del Rio and Huntsman, 2014; Martin et al., 2014). Broadly, and in concert with other factors, this results in circuit and network hyperexcitability (Contractor et al., 2015).

Interestingly, our group has observed periods of homeostatic changes in inhibition in the BLA of *Fmr1*KO mice corresponding with sensitive time periods in the developmental emergence of fear-learning (Vislay et al., 2013). These distinct changes in inhibitory neurotransmission suggest that amygdala-based behaviors may be altered in the *Fmr1*KO mouse early in development. During the period of inhibitory synaptic development (P10–P21) (Huntsman and Huguenard, 2000), our group has identified dramatic reductions in inhibitory neurotransmission from birth to P10. Between P14 and P16, a transient period of enhanced inhibitory function emerges (Vislay et al., 2013). Interestingly, these inhibitory changes occur in the absence of isolated changes in excitatory synaptic function, but rather occur in the context of increased feed-forward excitation. As such, at this developmental time point, FFI is observed to be broadly normalized. However, this is followed by a return to depleted levels of inhibition by P21. However, whether this period of enhanced inhibitory function represents a precocious maturation of GABAergic neurotransmission or a homeostatic response to hyperexcitability in early life remains to be definitively determined.

Our hypothesis about this phenomenon is that inhibitory changes during this time period represent inadequate homeostatic compensation and contribute to pathologic plasticity and behavioral outcomes. Indeed, our studies at P14 demonstrate that early life bidirectional changes in plasticity are dependent on the state inhibition. For instance, at P14, enhanced LTP is observed in the absence of GABA receptor blockade, whereas enhanced LTD is observed in presence of GABA receptor blockade suggesting that pathologic plasticity exists at baseline, but further driving excitation has the opposite effect. However, at P21, a marginal increase in LTP is observed in *Fmr1*KO animals at baseline, but under conditions of GABA receptor blockade, this LTP magnitude is diminished from the wild type (WT) baseline suggesting that as early as P21 plasticity in the FXS BLA is demonstrably reduced.

Future studies are warranted to characterize these changes. Indeed, one major driver of CP plasticity that has been understudied in FXS is neuromodulation. To our knowledge, few studies have focused on the canonical neuromodulators of CP plasticity and fear-learning in the *Fmr1*KO BLA, despite neuromodulation strategies (in particular, serotonin receptor modulators) showing promise for reversing behavioral deficits for FXS, ASDs, and NDDs more broadly (Armstrong et al., 2020; Lee et al., 2021; Jeon et al., 2022; Kim et al., 2022).

Similar to studies in human patients with FXS, studies examining fear learning in rodent models of FXS have produced conflicting results. *Fmr1*KO mice have been found to exhibit higher levels of hyperarousal and anxiety when interacting with novel conspecifics (McNaughton et al., 2008). However, studies employing classic Pavlovian fear-conditioning paradigms have identified decreases in contextual and conditioned freezing episodes suggesting deficiencies in both hippocampal and amygdala-based associative learning (Paradee et al., 1999). Indeed, these findings have recently been reproduced in a rat model of FXS (Fernandes et al., 2021). Further, despite the prevalence of amygdala-based disorders in human patients with FXS, mouse models of FXS demonstrate deficiencies in LTP in the circuits responsible for fear-learning (Zhao et al., 2005; Suvrathan et al., 2010). One possible explanation for these disparate findings is that plasticity and associative learning defects manifest differently at different ages. In our own work, we have shown this to be the case as LTP in the BLA is transiently enhanced early in development (Svalina et al., 2022). Thus, understanding early life cellular and synaptic alterations, CP plasticity in the BLA and how deficits emerge and change over time in FXS and ASDs is of great importance for developing new therapies.

### Ontogeny of fear-learning in the rodent

The ontogeny of amygdala-dependent fear-learning has been well characterized and involves a number of characteristic changes in the BLA and fear across ontogeny (Moriceau and Sullivan, 2005). At birth, altricial rodents depend on olfaction for orientation and navigation, maternal attachment, and nursing to receive the caregiving necessary for survival (Teicher and Blass, 1977; Galef and Kaner, 1980; Leon, 1992; Landers and Sullivan, 1999; Sullivan et al., 2003). Whereas the rodent visual and auditory systems develop later in life (around P14) (Blatchley et al., 1987; Shen and Colonnese, 2016). From birth to P10, pups do not exhibit amygdala-dependent fear learning (Sullivan et al., 2000a), but show robust olfactory learning necessary for their survival associated with a sensitive period for attachment learning and learning the maternal odor (Pedersen et al., 1982; Shen and Colonnese, 2016). However, at this developmental time point, pups display similar reward value to both appetitive and aversive stimuli to support attachment learning (Camp and Rudy, 1988; Perry et al., 2017). Indeed, rodent pups will exhibit an odor preference for a conditioned odor even when the odor is paired with a mild tail shock or milk, using a learning circuit involving the olfactory bulb and olfactory cortex, as well as failure to recruit the amygdala (Moriceau and Sullivan, 2006; Mukherjee et al., 2017; Oruro et al., 2020). Olfactory bulb NE is necessary and sufficient for associative olfactory learning in rodent pups and has been postulated to be essential for attachment learning (Sullivan et al., 1991, 2000b; Sullivan, 2001). It should be noted that pups during the first week of life can learn fear and avoidance with high shock or malaise, although learning depends upon the olfactory system and high levels of NE, and not the amygdala (Moriceau et al., 2009; Raineki et al., 2009). However, during this developmental epoch of quiescent amygdala function, the BLA is especially susceptible to early life adversity with enduring effects seen throughout the lifespan (Lukkes et al., 2012; Callaghan et al., 2019). Indeed, repeated adversity, such as maternal separation or maltreatment of rat pups in the first week of life results in a non-linear developmental trajectory of hyperactivity in the amygdala and enhanced contextual and conditioned fear-learning in later-life (Diehl et al., 2014; Pattwell et al., 2016; Junod et al., 2019). But amygdala deficits are also evident in infancy as reflected in precocial emergence of amygdala-dependent fear due to amygdala excitability, reduction of BLA axo-somatic synapses formed by parvalbumin cells, and perineuronal net and engagement of intracellular molecular events associated with plasticity (Ehrlich and Rainnie, 2015; Santiago et al., 2018; Opendak et al., 2019). Further, repeated stress or exogenous administration of corticosterone within a social context is considered important in the initiation of this altered developmental trajectory (Raineki et al., 2019; Opendak et al., 2020).

From P10 to P15, a transitional sensitive period emerges in which normal adult-like responses to odor-cued amygdala-dependent fear conditioning are evident (Sullivan et al., 2000a). Interestingly, social context is capable of blocking pup fear responses during this time (Barr et al., 2009). For instance, odor-cued fear conditioning conducted in the presence of the maternal dam suppresses fear acquisition in the rodent pup such that the pup will instead demonstrate an odor preference for the conditioned odor (Shionoya et al., 2007). This has been replicated in young children, illustrating a potential cross-species feature of infant attachment learning (Tottenham et al., 2019), with both species associated with decreasing stress hormone levels and attenuation of the amygdala, which wanes by adolescence (Gee et al., 2014). The rodent research has further defined the importance of NE and dopamine in this process (Barr et al., 2009; Opendak et al., 2019, 2021). These studies suggest that at this developmental time point the attachment and avoidance circuits exist in a delicate balance readily switched by maternal presence. By P16 infant rats have a more mature functioning of the amygdala-depended fear emerges and maternal presence plays a limited role in modulating or only slightly attenuating avoidance and fear, potentially through a decrease in maternal modulation of dopamine and maturing amygdala (Upton and Sullivan, 2010; Opendak et al., 2021). These unique age-specific features of infant learning are due to age-specific mechanisms illustrating that the developing brain is not an immature version of the adult brain and highlights how the environment impacts its development.

Emerging data also illustrates that developmental disorders, potentially associated with genetic abnormalities in *Fmr1*KO mice and rodent models of FXS can also impact the amygdala and disrupt the developmental trajectory of the amygdala. For example, in contrast to previous studies in aged mice, our most recent work in mouse pups has shown that LTP and fear-learning are briefly enhanced at the infant P14 developmental time point. Interestingly, this pathologic LTP and fear-learning is dependent on the state of inhibition and can be ameliorated with early-life 4,5,6,7-tetrahydroisoxazolo [5,4-c]pyridin-3ol (THIP) intervention at this time point. Thus, it is essential to better study developmental changes in NDDs to not only understand how to treat but also when to treat and the importance of defining age-specific interventions.

## Future directions

**FIGURE 1:**
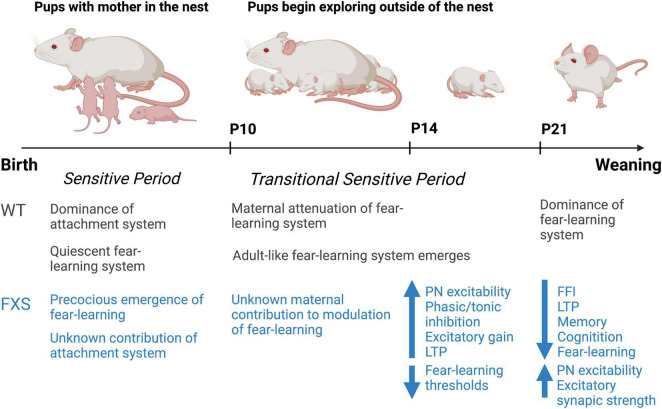
Summary timeline of observed changes in early development in the *Fmr1*KO mouse compared to the wild-type rodent. Figure adapted from previous work by Regina Sullivan.

We integrate the current state of the literature as shown in Figure 1. The broad spectrum function of FMRP enables several avenues of dysfunction but also opens several potential therapeutic avenues. For instance, it remains unknown if the intrinsic properties of neurons are altered at the earliest post-natal time points. Similarly, it remains to be determined how circuit-level changes contribute to the maladaptive behavioral manifestations seen in FXS throughout early development. Further, how social and environmental factors impact the behavior in FXS is an interesting area worthy of future study. Though suggested by our own work and that of others, correlative circuit-level changes may underpin behavioral alterations but direct causation studies are certainly warranted. Indeed, the extended and enhanced period of plasticity observed in NDDs such as FXS likely holds the key to the discovery of specific changes at the circuit level (Meredith et al., 2012). A major challenge for the study of NDDs, like FXS, is the discrepancy between the onset of distinct molecular and synaptic pathophysiology and the appearance of symptoms. It may be the case, that the time at diagnosis may be too far beyond the CP of plasticity to provide the platform needed for circuits to be modified. Therefore, the identification of specific circuit-level changes during critical periods of brain development is of high importance for our understanding of the pathophysiology and treatment of FXS patients.

## Author contributions

MS and MH consulted on the original content. MS wrote the original draft. MS, RS, DR, and MH edited the final version. All authors contributed to the article and approved the submitted version.
